# Bone Marrow‐Derived Mesenchymal Stem Cells (BM‐MSCs) and Small Intestinal Submucosa (SIS) Hydrogel Composite Can Meliorate TNBS‐Induced Experimental Colitis Through Regulating Inhibiting Macrophage Polarization Toward the M1 Phenotype

**DOI:** 10.1155/mi/9968648

**Published:** 2026-05-30

**Authors:** Zikai Sun, Yan Zhou, Xiang Geng, Yu Gong, Fengdong Li, Shaohua Zhuang, Xiang Cao, Jin Huang, Jinjin Fu

**Affiliations:** ^1^ Department of Gastroenterology, The Changzhou No. 2 People’s Hospital Affiliated to Nanjing Medical University, Changzhou, China, njmu.edu.cn

**Keywords:** bone marrow mesenchymal stem cells (BM-MSCs), experimental colitis, macrophage polarization, small intestinal submucosa (SIS)

## Abstract

An imbalance of macrophage polarization exerts influence over inflammatory bowel disease (IBD) pathogenesis. To investigate how macrophage polarization influences IBD, we engineered a composite system consisting of small intestinal submucosa (SIS) hydrogel combined with bone marrow‐derived mesenchymal stem cells (BM‐MSCs). The therapeutic potential of this construct was assessed by examining its regulatory effects on macrophage polarization both in a 2,4,6‐trinitrobenzene sulfonic acid (TNBS)‐induced colitis mouse model and in RAW264.7 macrophages in vitro. Clone formation assay was executed to identify the influence of MSC‐SIS‐conditioned medium on the proliferation of RAW264.7. CD206 and CD86 were measured by flow cytometric. Quantitative real‐time PCR (qRT‐PCR) was conducted to evaluate cytokine gene expression. Experimental colitis was induced in mice using TNBS. Disease severity was assessed by calculating the clinical disease activity index (CDAI). Colonic tissues were subjected to histological and morphometric analyses, and serum levels of inflammatory cytokines were quantified using enzyme‐linked immunosorbent assay (ELISA). Data indicated that the BM‐MSCs and SIS hydrogel composite significantly promoted Raw264.7 proliferation. Compared with the lipopolysaccharide (LPS) + MSCs group, treatment with the combined MSCs and SIS hydrogel construct demonstrated a more pronounced inhibitory effect on M1 phenotype the autophagy of LPS‐induced RAW264.7 in vitro; what is more, BM‐MSCs and SIS hydrogel greatly inhibited cytokine mRNA levels in LPS‐stimulated Raw264.7 cells compared with treatment with MSCs solely. In addition, the results implied that the mice in TNBS + MSC + SIS group had alleviated colitis compared with the TNBS + MSCs group, and the interleukin (IL)‐1β and tumor necrosis factor‐α (TNF‐α) levels in the serum also decreased. In conclusion, these results indicate a better wound healing effect of SIS hydrogel on BM‐MSCs through promoting cell regeneration and inhibiting macrophage polarization towards the M1 phenotype in a TNBS‐induced experimental mouse model.

## 1. Introduction

Inflammatory bowel disease (IBD) is a long‐term, idiopathic condition marked by persistent inflammation of the gastrointestinal tract. It primarily includes two main forms: Crohn’s disease (CD) and ulcerative colitis (UC). [1, 2]. In IBD, the inflammation of the intestinal mucosa may show various symptoms, such as bloody stools, diarrhea, weight loss, abdominal pain, and the influx of neutrophils as well as macrophages, which generate proteolytic enzymes, cytokines, and free radicals that lead to inflammation and ulceration [3, 4]. However, as the understanding of precise pathophysiology underlying IBD is still limited, there is no cure for IBD yet. Recent strategies only target the control of symptoms, with limited success in modifying the underlying disease progression or addressing its root causes. [5–8]. Patients with IBD suffer from spontaneous increased risks of thrombotic events, skeletal disorders, and intestinal cancers, including small intestinal malignancies, colorectal dysplasia, colorectal cancer, and potentially a minor higher risk of lymphoma [6, 8–11]. Thus, IBD imposes burdens in terms of health and economics on communities around the globe and lowers patients’ quality of life to a substantial extent [12].

The exact mechanisms responsible for the development of IBD remain incompletely understood. Evidence from previous research suggests that the disease is linked to abnormal regulation of the immune system [13]. Some of the major factors that lead to the pathogenesis of IBD include intestinal epithelial barrier disruption and macrophage dysregulation. In the regulation of tissue repair, the primary effector cells are macrophages, and the microenvironment of the injured sites can mediate the reprogramming of the macrophage phenotype [14–19]. What is more, they play a vital role in regulating intestinal microenvironment homeostasis, which can aggravate injured colon tissue inflammation by means of phenotypic polarization, and are closely relevant to IBD pathogenesis [20]. Macrophages are generally divided into two main phenotypes: M1 and M2. M1 macrophages are primarily involved in proinflammatory immune activity, whereas M2 macrophages contribute to anti‐inflammatory functions and tissue repair processes. [21, 22].Macrophages play a significant role in IBD by modulating their polarization toward either a proinflammatory (M1) or anti‐inflammatory (M2) phenotype in response to different environmental signals. The polarization of M1 macrophages implies that it responds to lipopolysaccharides (LPSs) and toll‐like receptor signaling, secreting cytokines that facilitate inflammation (e.g., interleukin [IL]‐1β, IL‐23, IL‐12, IL‐6, and tumor necrosis factor‐α [TNF‐α]). It not only indicates enhanced antigen‐presenting activity but also proinflammatory phenotypes. Meanwhile, they can eliminate the infections resulting from bacterial, viral, and fungal factors [23, 24]. Reversely, stimulation with IL‐4 and IL‐13 promotes macrophage differentiation toward the M2 phenotype, which functions to suppress or attenuate immune responses by secreting high‐level cytokines that prevent inflammation, including arginase‐1, IL‐10, CCL24, CCL17, CCL22, and TGF‐β [22, 24–26]. Such balance between the two subtypes is of great significance in eliminating inflammation in infection and injury and the repair processes by means of the suppression of proinflammatory immune responses and tissue repair regulation as well as angiogenesis [27, 28].

As multipotent stem cells derived from the mesoderm, they possess multipotent differentiation abilities along with a strong capacity for self‐renewal. During recent years, some reports have shown that mesenchymal stem cells (MSCs) transplantation facilitates recovery in IBD by enhancing the regeneration of the intestinal mucosal epithelium. [29–31]. MSCs, which are heterogeneous, secrete various substances that enable immunity regulation, inflammation reduction, and tissue repair promotion through changes in the microenvironment. Previous research has demonstrated that preconditioning MSCs with miRNAs, IL‐1β, or IFN‐γ enhances their ability to modulate macrophage activity and reduce inflammatory reactions. Further, the functions of MSCs in anti‐inflammation and repair rely on their microenvironment and state of activation [32]. Some previous studies showed that MSCs administered intravenously could attenuate the asthma phenotypes of chronic asthma via the modulation of macrophages. Identification of M2 macrophage subtypes suggested that exposing to MSCs is able to transform the phenotype and functions of macrophages [33]. Based on their potential in immunomodulation and tissue regeneration, MSCs are increasingly recognized as a viable therapeutic option for managing IBD. Previous studies on MSCs indicated that long‐term survival and proper integration with the host tissue were not observed following postadministration [34].

To some extent, for IBD, stem cell therapies have the limitations of low rates of cell retention and engraftment after transplantation [35]. MSCs transplantation has been observed as a great therapy for IBD and similar immune disorders [36, 37]; however, the efficacy of such therapy is reflected by the count of posttransplantation surviving cells [38]. Unsatisfactory cell engraftment and retention after transplantation serve as the major reasons that hinder the promotion of stem cell‐based therapies [37].

Porcine small intestinal submucosa (SIS), a naturally derived extracellular matrix (ECM), is harvested from the jejunal section of the porcine small intestine. It is found that SIS contains collagens, various bioactive factors (TGF‐β, VEGF, and IGF‐1), fibronectins, glycoproteins, chondroitin sulfates, and proteoglycans, as well as glycosaminoglycans [39–41]. SIS, when combined with its cytokines, can offer an effective scaffold for cellular ingrowth and proliferation, which facilitates the migration of cells to the inside of the implant, enabling a quick replacement of SIS by endogenously‐produced tissues [42, 43]. SIS, a key dECM biomaterials, demonstrates substantial clinical potential in soft tissue injury repair owing to its well documented bioactivity, nonimmunogenicity, and controllable biodegradability [44]. SIS biomaterials have been extensively used in many species for tissue remodeling and repair, such as in urethral and vaginal prolapse repair [45], skull base reconstruction [46, 47], upper airway tissue remodeling [48], full‐thickness skin defects [49], tympanic membrane repair [50], and cardiovascular diseases [51]. SIS hydrogel is derived from porcine SIS. Furthermore, lyophilized dECM powder can be partially digested into a thermo‐responsive and pH‐sensitive hydrogel (denoted dECMh), which can be directly placed to the injured sites [14–17] or deliver the cells. SIS hydrogel better replicates the native microenvironment of intestinal organoids, thereby providing a more supportive setting for their growth and development [52].

MSC‐based therapies are very promising in the treatment of IBD. With the aim of improving and enhancing the medicinal benefits of MSCs, we carried out an investigation to identify if the SIS‐based injectable hydrogel and bone marrow‐derived MSCs (BM‐MSCs) composite cotransplantation could ameliorate 2,4,6‐trinitrobenzene sulfonic acid (TNBS)‐induced experimental colitis via the regulation of inhibiting macrophage polarization towards the M1 phenotype.

## 2. Methods and Materials

### 2.1. Ethics Statement

The care, operation, feeding, and treatment of test animals were all carried out as per the institution’s guidelines. All the experiments received approval from the Animal Research Ethics Committee of Nanjing Medical University in China (Approval Number 2021DZGKJDWLS‐00143).

### 2.2. Preparation of SIS Hydrogel

In order to get SIS, we took the porcine jejunum from a market pig and decellularized it by means of the method used by Badylak [53]. Briefly, the muscular and serosal layers, along with residual adipose tissue, were mechanically removed from the porcine jejunum. We used 0.1% peracetic acid and a freeze dryer to disinfect and lyophilize the SIS, respectively. Then, a freezer mill was used to crush the lyophilized SIS in liquid nitrogen into 20 µm powder.

SIS hydrogel was prepared based on the aforesaid methods [54, 55]. Specifically, we suspended the pulverized SIS in porcine pepsin (1 mg/mL, Sigma–Aldrich) prepared in 0.01 mol/L HCl and continuously stirred at 60 rpm for 48 h at room temperature. The resulting digest was adjusted to pH 7.4 with 0.1 mol/L NaOH, followed by lyophilization to obtain a soluble SIS matrix. The dried product was sterilized by γ‐irradiation and milled again to yield the final SIS powder, which was subsequently reconstituted in 20% (w/v) phosphate‐buffered saline (PBS).

### 2.3. Cell Culture and CM Collection

RAW264.7 murine macrophages were used for the cell study. The RAW264.7 cell line was kindly provided by the Laboratory of General Surgery at The Changzhou No. 2 People’s Hospital, affiliated with Nanjing Medical University. Cells were maintained in Dulbecco’s modified Eagle medium (DMEM; Gibco) supplemented with 10% fetal bovine serum (FBS; Gibco). We dissolved LPSs (Sigma) by using sterile distilled water to prepare a 1 mg/mL stock solution and further diluted it to reach 100 ng/mL in the cell culture media.

BM‐MSCs have been isolated, cultured, and identified (https://www.techscience.com/biocell/online/detail/19522). In an effort to make SIS gel‐coated plates for in vitro culture of BM‐MSCs, we poured 1 mL of the SIS suspension into a 35 mm culture dish and incubated at 37°C overnight to form the gel.

The conditioned medium from SIS (SIS‐CM), BM‐MSCs with SIS gel composite (MSC + SIS‐CM), and MSCs (MSC‐CM) were collected, respectively, after 24‐h incubation with MSCs/SIS hydration/MSCs + SIS for in vitro experiments, as previously described [56]. The medium was filtered through a 0.45‐μm membrane (Millipore), then aliquoted and stored at –80°C for later use.

The RAW264.7 cells were divided into five groups: LPS + SIS, LPS + MSCs, LPS + MSCs + SIS, LPS, and control. In the LPS group, LPS + MSCs group, LPS + SIS group, and LPS + MSCs + SIS group, cells were handled with 100 ng/mL LPS for 24 h; in the LPS + MSCs + SIS group, cells were handled with MSC + SIS‐CM; in the LPS + SIS group, cells were cultured with SIS‐CM; and in the LPS + MSCs group, cells were handled with MSC‐CM.

### 2.4. Clone Formation Assay

The RAW264.7 cells (200 cells/well) in various groups were transferred to 6‐well plates and cultivated in a carbon dioxide incubator with specific conditioned medium. After about 14‐day cultivation, we fixed these cells using 4% paraformaldehyde and stained with 0.1% crystal violet (Beyotime, Beijing, China). Thereafter, we photographed the colonies by an inverted microscope (MTX Lab Systems, Bradenton, FL, USA) and calculated the number with Image J.

### 2.5. Flow Cytometric Analysis

In combination with IL‐4 (Proteintech, Wuhan, China), we utilized LPS (Sigma) to establish the models for M1 and M2 polarization in the RAW264.7 cells. In the M1 polarization model, LPS 100 ng/mL was used, while 20 ng/mL IL‐4 was applied in the M2 polarization group. We used CD86 to mark M1 macrophages and identified M2 macrophages with CD206. After the indicated treatment, we detached RAW264.7 cells with 0.25% trypsin‐EDTA (Gibco) for 20–30 s, then centrifuged at 1000 rpm for 5 minutes. The supernatant was discarded, and cells were washed twice with PBS, then re‐suspended in PBS. Cell surface markers, CD86‐APC (Multi Sciences Biotech Co., Ltd., China) and CD206‐PE (AtaGenix, Wuhan, China), were used for cell labeling on ice for 30 minutes in darkness. Then we washed the cells twice and resuspended them in 200 μL of PBS ahead of conducting the analysis. Subsequently, flow cytometry was then conducted using a flow cytometer (Beckman Coulter).

### 2.6. RNA Was Extracted, and Quantitative Real‐Time PCR (qRT‐PCR) Was Performed

Total RNA was extracted from RAW264.7 macrophages with Trizol reagent (Vazyme Biotech Co., Nanjing, China). Reverse transcription was carried out using HiScript ⅡQ RT SuperMix for qPCR (Vazyme Biotech Co., Nanjing, China). Real‐time PCR was conducted with Green Master Mix (low ROX Premixed) (Vazyme Biotech Co., Nanjing, China) following the manufacturer’s instructions. Gene expression analysis for mouse *IL10*, *TNFα*, *IL6*, and *Actin β* (all from Sangon Biotech, China) was performed via qRT‐PCR. The gene‐specific primer sets can be found in Table 1. We normalized the expression levels of the genes to actin.

**Table 1:** Primers for real‐time qPCR.

Genes	Forward primer (5′–3′)	Reverse primer (5′–3′)
*Actin β*	*TGGAATCCTGTGGCATCCATGAAAC*	*TAAAACGCAGCTCAGTAACAGTCCG*
*IL6*	*AAGTCCGGAGAGGAGACTTC*	*TGGATGGTCTTGGTCCTTAG*
*TNFα*	*GATTATGGCTCAGGGTCCAA*	*ACAGAGGCAACCTGACCACT*
*IL10*	*AGTGGAGCAGGTGAAGAGTGATTT*	*CTATGCAGTTGATGAAGATGTC*

### 2.7. Animals and Induction of TNBS‐Induced Colitis

Male BALB/c mice with an age of 8–10 weeks were purchased from CAVENS.LA technology (Changzhou, China). Those mice used in the experiments had a body weight of 20–22 g. The mice were placed at 22–24°C under 12‐h light/dark cycle and had ad libitum access to food and water. Following an overnight fast, the animals were anesthetized via ether inhalation.

TNBS modeling in mice was established following the method described by Morris [57]. Briefly, we dissolved 2.5 mg of TNBS acid (Sigma–Aldrich Corp) in 50% ethanol and administered it intrarectally to a position that was 4 cm proximal to the anus (a total volume of 100 μL) by using a lubricated silicone catheter. MSC therapy was given to BALB/c mice 3 h after TNBS administration, when tissue damage reached the peak [58]. MSCs (at a dose of 1 × 10^6^ cells)/SIS hydrogel/MSCs + SIS hydrogel were administered via enema in 100 μL of sterile PBS. After the treatment, we weighed and monitored the mice on a daily basis. On Day 7 after the administration of TNBS, the animals were culled by means of stunning and exsanguination. The mice (*n* = 25) were randomly grouped into four cohorts, with five animals each: the control group, TNBS group, TNBS + MSCs group, TNBS + SIS group, and TNBS + MSCs + SIS group.

### 2.8. Clinical Score and Evaluation of Disease Activity Index (DAI)

DAI and body weight [59], which are widely used to evaluate colitis, were measured every day after the occurrence of colitis. Changes in body weight were calculated as the percentage of weight loss compared to baseline.

### 2.9. Histology and Morphometric Analyses

After the mice were killed, colons were excised, colonic lengths were measured, and the general appearance and histology of the colonic mucosa were evaluated. A section of the distal colon was fixed in 10% buffered formalin for paraffin embedding. The tissue sections were then dyed with H&E as per standard protocols.

### 2.10. Enzyme‐Linked Immunosorbent Assay (ELISA)

We collected blood samples from each of the mice and then separated and froze the serum at −80°C for future use. The serum levels of TNF‐α and IL‐1β were assessed with an ELISA kit (Camilo Bioengineering Co., Ltd., China) as per the manufacturer’s instructions.

### 2.11. Statistical Analysis

Statistical analyses were performed using GraphPad Prism (Ver. 8.0.2, GraphPad Software). Data are displayed in the form of average values ± SD. Differences between groups were assessed using the Student’s *t*‐test or ANOVA, with a *p*‐Value  < 0.05 considered statistically significant.

## 3. Results

**Figure 1:**
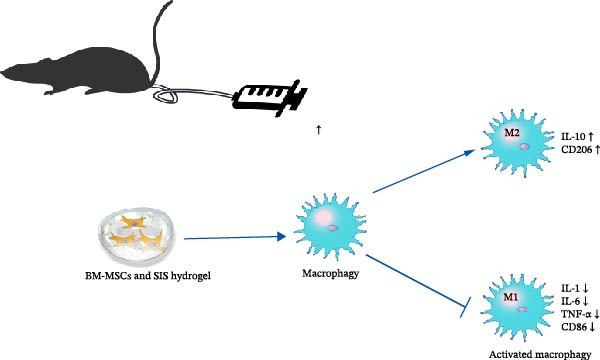
The potential mechanism of inflammatory suppression of the bone marrow‐derived mesenchymal stem cells (BM‐MSCs) and SIS hydrogel composite.

The mechanism underlying the therapeutic effects of SIS‐based injectable hydrogel and BM‐MSCs cotransplantation on TNBS‐induced colitis is illustrated in the accompanying figure (Figure 1).

### 3.1. Decellularization Process and Characterization of dECM Material

With a view to maintaining the structure of the protein on the basis of thorough decellularization, we modified a conjoint processing strategy in consideration of announced protocols [60]. The strategy mainly consists of the following parts: (i) tissue harvest and cleaning, which aimed to clean the external layer, mucosa, and mesentery; (ii) deoxyribonuclease I (DNase‐I) as well as sodium deoxycholate (SDC) treatment, which aimed to erase the cells and residual DNA; (iii) lyophilization as well as powder grinding; (iv) digestion with pepsin and HCl was carried out to prepare the pregel; (v) alterations in salinity, pH, and temperature initiated the gelation process. A qualitative analysis of the decellularized tissue was performed by staining the histological sections. The clearing of nuclei and other cell components was validated by H&E staining (Figure 2).

Figure 2The subparts show the process of SIS powder preparation and gelation, involving harvest of a small intestine from piglets that were newly killed; submucosa decellularization (A); lyophilization and powder grinding (C); the process involves sterilization, followed by digestion using pepsin and hydrochloric acid (HCl). The pH and salinity are then adjusted before the sample is incubated at 37°C to facilitate the gelation of the SIS powder (D); qualitative analysis of the decellularized small intestine tissue with hematoxylin and eosin (H&E) (B); (magnification, ×20).

**Figure:**
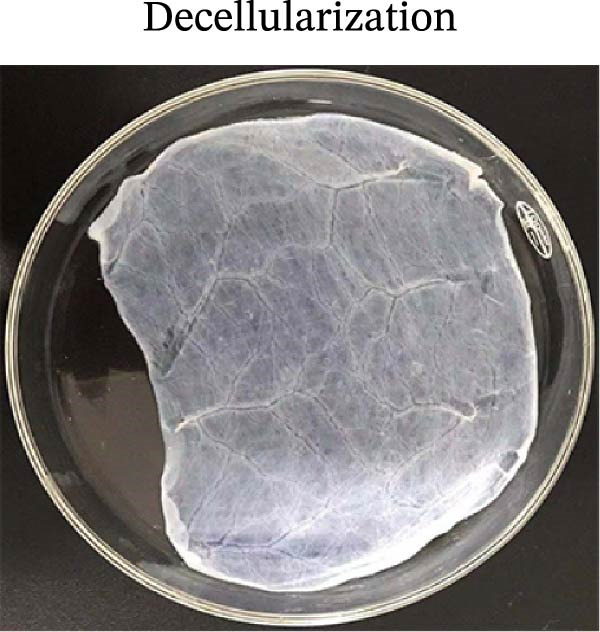
(A)

**Figure:**
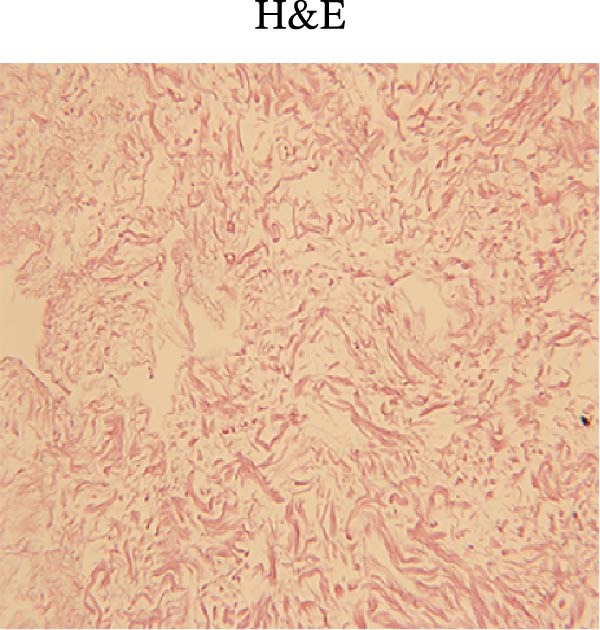
(B)

**Figure:**
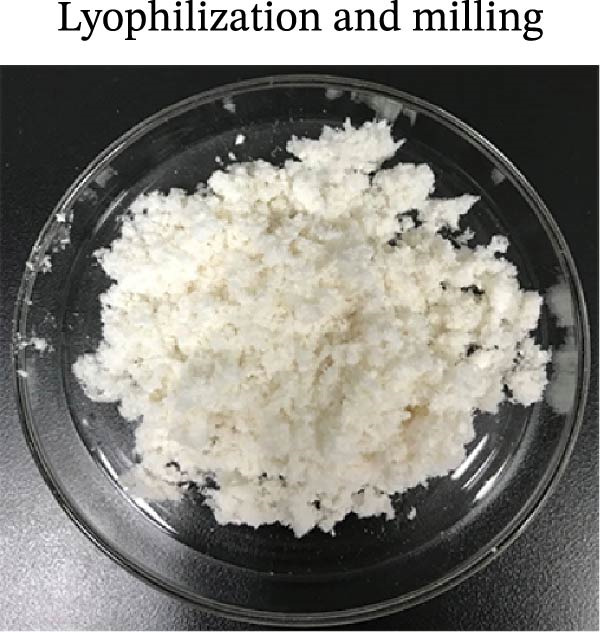
(C)

**Figure:**
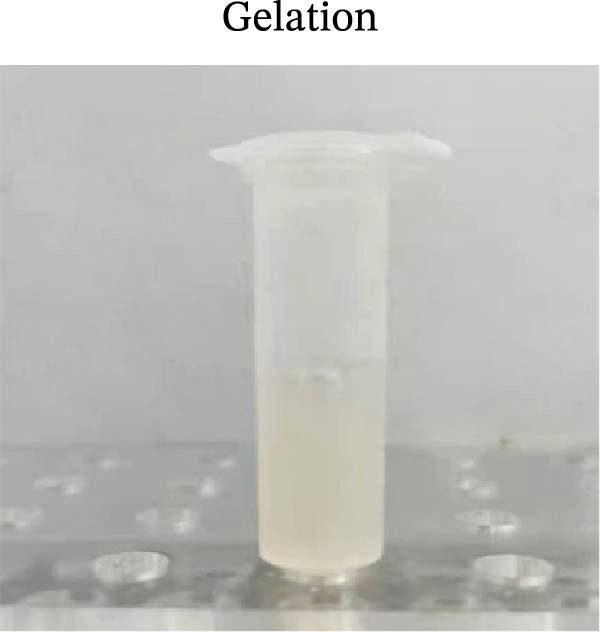
(D)

### 3.2. BM‐MSCs and SIS Hydrogel Composite Promoted Raw264.7 Cell Proliferation

The colony formation test, as an effective method, can be used to measure the proliferation ability of cells. The adherent cells might fail to proliferate and produce clones, while the cells that form clones must be adherent and proliferative. Based on the results of clone formation assay, BM‐MSCs/SIS hydrogel could promote the cell clone formation by comparison with the control group. Nevertheless, the BM‐MSCs and SIS‐CM group showed a much better effect in promoting cell clone formation, leading to an increased number of colonies compared with other groups (Figure 3A,B). The above outcome demonstrated that the BM‐MSCs and SIS hydration composite could dramatically enhance the reproduction ability of Raw264.7 cells in contrast to the method where they were co‐cultured with BM‐MSCs/SIS.

Figure 3Effects of BM‐MSCs‐SIS culture media on Raw264.7 proliferation (A). The four observations of stained Raw264.7 cells were estimated with Image J software (B). ^∗^ *p*  < 0.05,  ^∗∗^ *p*  < 0.01 (control group vs. MSCs/SIS/MSCs + SIS group); # *p*  < 0.05 (MSCs group vs. MSCs + SIS group); ns, no statistical significance. All values are provided in the form of mean ± standard deviation (SD).

**Figure:**
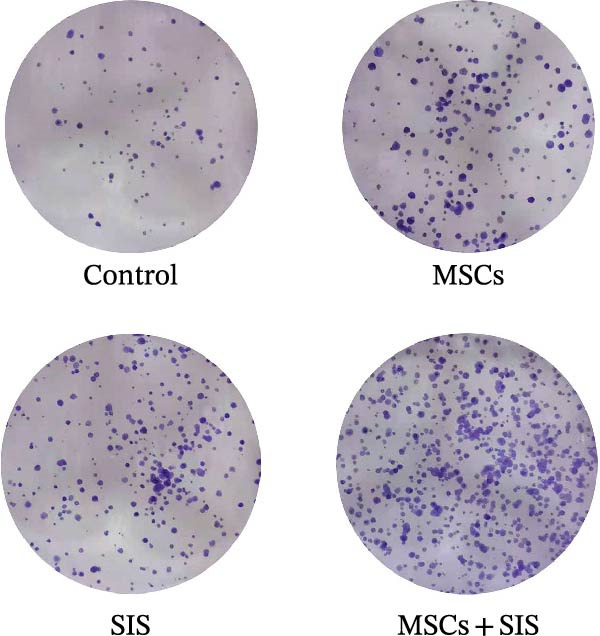
(A)

**Figure:**
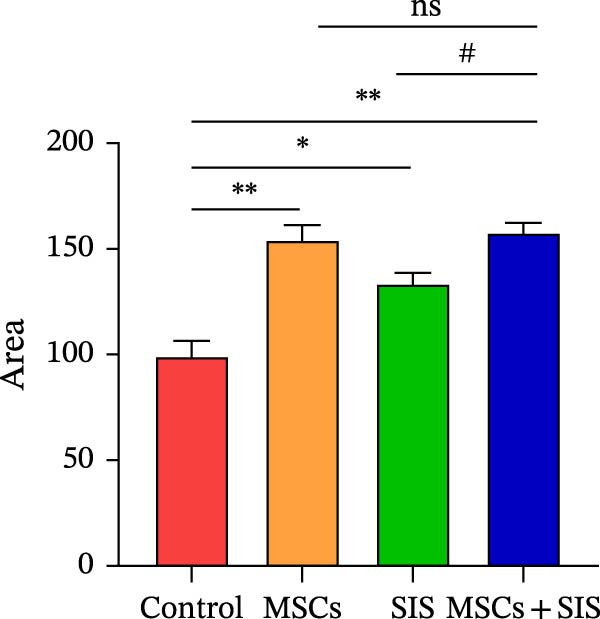
(B)

### 3.3. Effect of BM‐MSCs and SIS Hydrogel on Macrophage Polarization

In the process of responding to various local stimuli or diversified pathophysiological circumstances, macrophages might be converted to the M1 subtype, which promotes inflammation, or M2 subtype, which inhibits inflammation. In the course of inflammation, macrophages are mainly identified in M1‐type clusters, while M2 ones deliver protection to guarantee the intestinal functions’ homeostasis and immune responses. With consideration of the ability of BM‐MSCs to regulate macrophage polarization, we used flow cytometry to identify the macrophage phenotypes so as to determine the effect of the BM‐MSCs and SIS hydrogel composite on the RAW264.7 cells’ polarization. In this study, CD206 and CD86 were used as the specific markers for the M2 and M1 phenotypes, respectively (Figure 4).

Figure 4Flow cytometry results of CD86 (A) (a marker for M1 macrophage) and CD206 (C) (a marker for M2 macrophage) expression in RAW264.7 cells. In comparison with the LPS/IL4‐treated group:  ^∗∗^ *p* < 0.01; as opposed to the LPS/IL4 + MSCs + SIS group:  ^∗∗^ *p* < 0.01. In comparison with the LPS/IL4+MSCs‐treated group: ^##^ *p* < 0.01 (B, D). ns, no statistical significance; all values are provided as mean ± SD.

**Figure:**
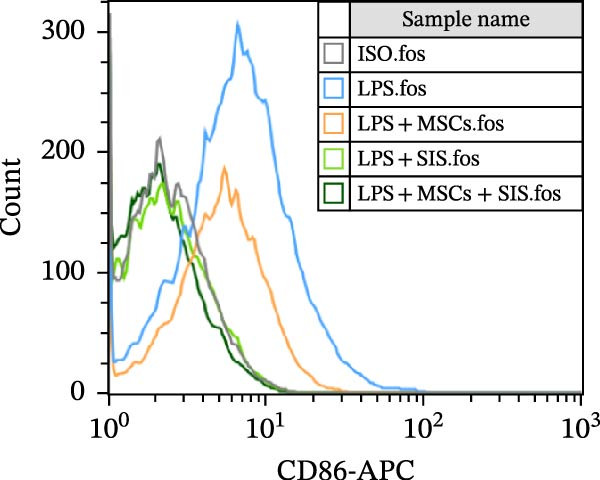
(A)

**Figure:**
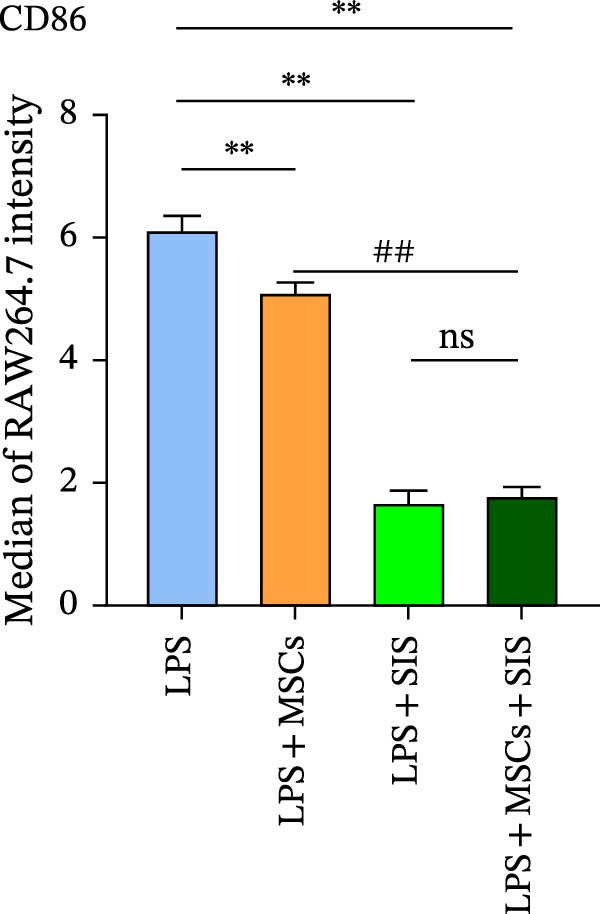
(B)

**Figure:**
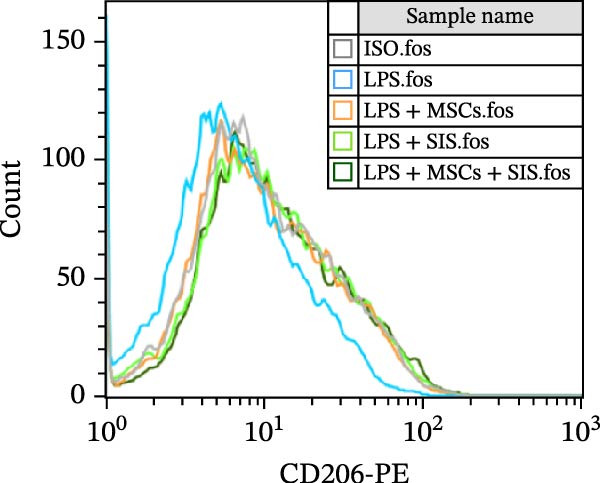
(C)

**Figure:**
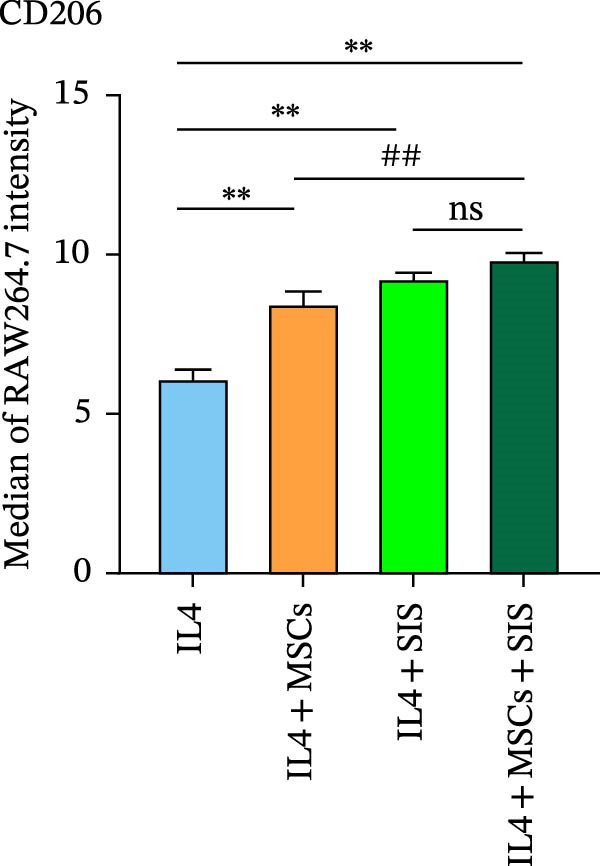
(D)

Subsequently, the culture medium of BM‐MSCs/SIS/BM‐MSCs‐SIS was used to arouse RAW264.7, and the observations found fewer CD86‐positive cells in the LPS + MSCs/LPS + SIS/LPS + MSCs + SIS group compared to the LPS‐only group (Figure 4A,B, ^∗∗^ *p*  < 0.01). CD86 expression was greater in the LPS + MSCs group compared to the LPS + MSCs + SIS group (^##^ *p*  < 0.01), although this difference was not statistically significant, which means that BM‐MSC‐SIS‐CM had a better effect in inhibiting CD86 expression than BM‐MSC‐CM alone.

CD206 expression increased in the IL4 + MSCs/IL4 + SIS/IL4 + MSCs + SIS group compared with IL4 group (Figure 4C,D,  ^∗∗^ *p*  < 0.01). BM‐MSCs/SIS/BM‐MSCs + SIS enhanced the CD206 expression in the IL4induced RAW264.7 cells (Figure 4C,D). In comparison, higher CD206 expression was identified in the IL4 + MSCs + SIS group compared to the IL4 + MSCs group (^##^ *p*  < 0.01), though this difference was not statistically significant between the LPS + MSCs + SIS group and LPS + SIS group. Based on these findings, we concluded that BM‐MSC‐ SIS‐CM had a better effect in promoting CD206 expression than BM‐MSC‐CM alone. Collectively, these findings showed that the BM‐MSCs and SIS hydrogel composite could better inhibit CD86 expression, the specific marker of M1 phenotype of macrophage; meanwhile, higher expression of CD206 was found in the BM‐MSC + SIS group compared to the MSC group, which implied that SIS hydrogel could better uplift the expression of M2 phenotye in BM‐MSCs treated cells.

### 3.4. Effects of the BM‐MSCs and SIS Hydrogel Composite on Inflammatory Cytokine mRNA Expression in LPS‐Induced RAW264.7 Macrophages

In an attempt to probe into the anti‐inflammatory activity of the BM‐MSCs and SIS hydrogel composite, we formulated an RAW 264.7 cell inflammation model induced by LPS in the first place.

To examine the expression levels of several proinflammatory cytokines, we conducted real‐time qRT‐PCR on the LPS‐only, the control, the LPS + SIS, LPS + MSCs, and LPS + MSCs + SIS groups. The mRNA expression levels of TNF‐α, IL‐6, and IL‐10 in each group are shown in Figure 5A–C. Significantly higher levels of *IL-6* and *TNF-α* mRNAs were identified in the LPS‐only group compared to the control group (*p*  < 0.01).BM‐MSCs/SIS sharply weakened the secretion of the two cytokines that facilitate inflammation compared to the LPS‐only group (*p*  < 0.01); and greatly lower levels of *IL6* and *TNFα* expression were observed in the LPS + MSCs + SIS group in contrast to the LPS + MSCs group. However, no significant difference was found between the TNBS + SIS and TNBS + MSCs + SIS groups (*p*  > 0.05), which means that SIS hydrogel had a better effect in decreasing the *IL6* and *TNFα* expression levels than BM‐MSCs‐CM alone.

Figure 5Levels of gene expression of (A) *TNFα*, (B) *IL6*, and (C) *IL10* in RAW264.7 cells from various treatment groups (*n* = 3, mean ± SD).  ^∗^ *p*  < 0.05,  ^∗∗^ *p*  < 0.01 (control group vs. LPS group); ^#^ *p*  < 0.05, ^##^ *p*  < 0.01 (LPS group vs. LPS + MSCs/SIS/MSCs + SIS group); ^ɑ^ *p*  < 0.05, ^ɑɑ^ *p*  < 0.01 (LPS + MSCs + SIS group vs. LPS + MSCs/SIS group); ns, no statistical significance.

**Figure:**
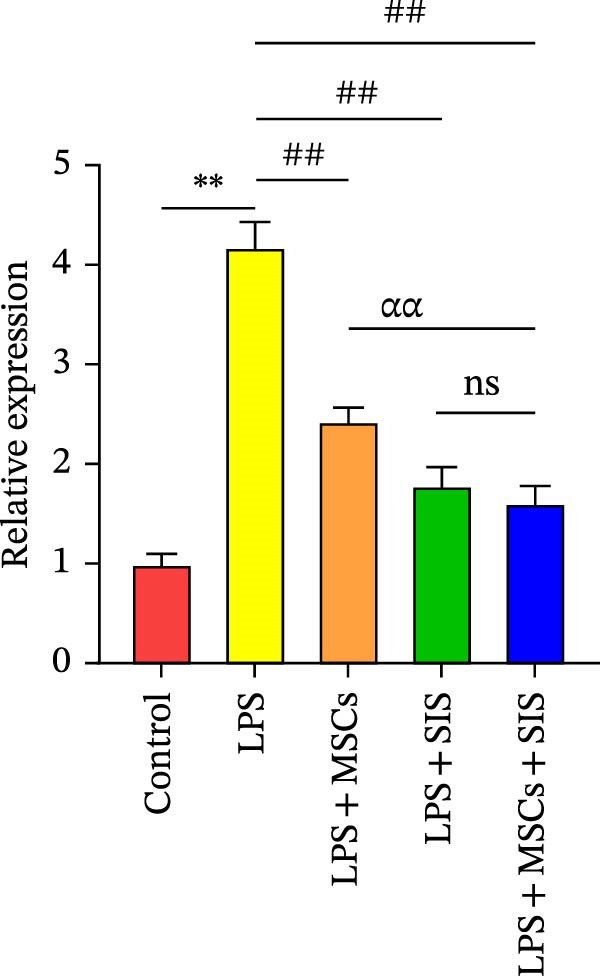
(A)

**Figure:**
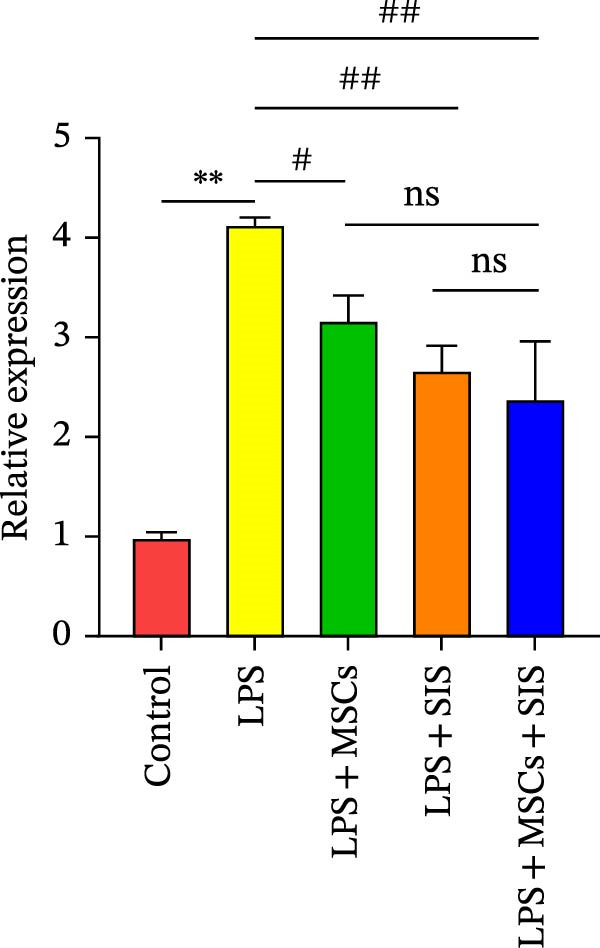
(B)

**Figure:**
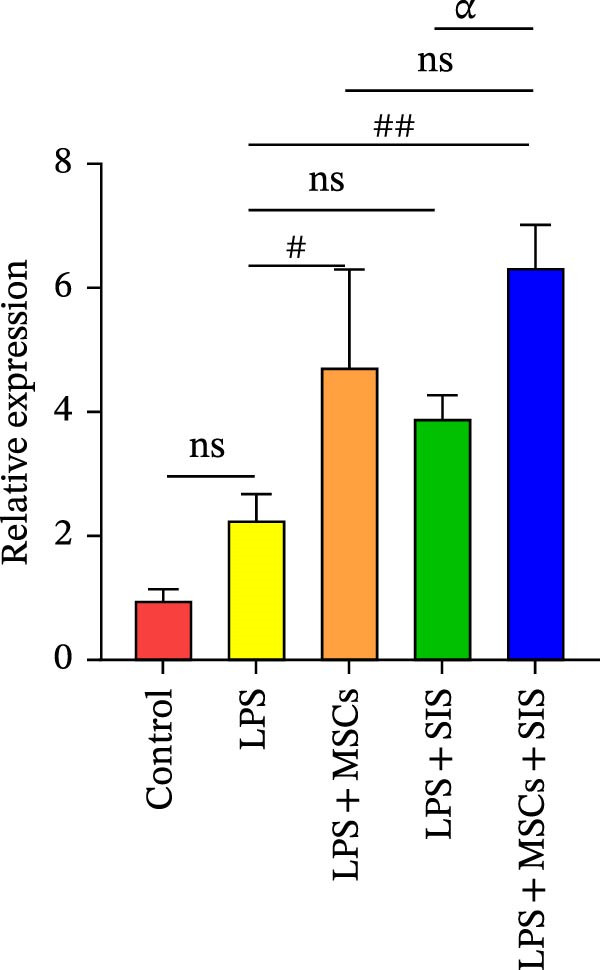
(C)

Meanwhile, significantly higher levels of mRNAs of *IL-10* were identified in the LPS + SIS/MSCs + SIS group in contrast to the LPS‐only group (*p*  < 0.01). Moreover, *IL10* titers were higher in the LPS + MSCs + SIS group than in the LPS + MSCs/SIS group.

As *IL6* and *TNFα* serve as M1 markers, and *IL10* is an M2 marker of macrophages, we preliminarily inferred that the MSCs and SIS hydrogel composite greatly influenced their anti‐inflammatory activities in decreasing proinflammatory cytokine *IL6* and *TNFα* expression and increasing anti‐inflammatory cytokine *IL10* mRNA expression by enhancing M2 macrophages and decreasing M1 macrophages.

### 3.5. The BM‐MSCs and SIS Hydrogel Composite Ameliorates TNBS‐Induced Colitis in Mice

Furthermore, we built a TNBS‐induced colitis mice model to investigate BM‐MSCs and SIS hydrogel (Figure 6) for their potential effect against inflammation. Throughout the modeling process, mouse body weights were monitored, and stool conditions were observed at 19:00. On the 7th day, the mice were euthanized, and colon lengths were measured.

Figure 6Reduction in colitis severity after treatment with BM‐MSCs and SIS hydrogel. (A) Body weight measurements of mice in different groups. (B) Disease activity indexes (DAIs) for each group. (C) Representative images of the colon from normal mice, TNBS‐treated mice, TNBS‐MSC‐treated mice, TNBS‐SIS‐treated mice, and TNBS‐MSCs‐SIS‐treated mice. (D) Colon length measurements for each group.  ^∗∗^ *p* < 0.01 (LPS + MSCs vs. control); ^#^ *p* < 0.05, ^##^ *p* < 0.01(LPS vs. LPS + MSCs/SIS/MSCs + SIS); ^ɑ^ *p* < 0.05 (LPS + MSCs + SIS vs. LPS + MSCs/SIS); *n* = 5.

**Figure:**
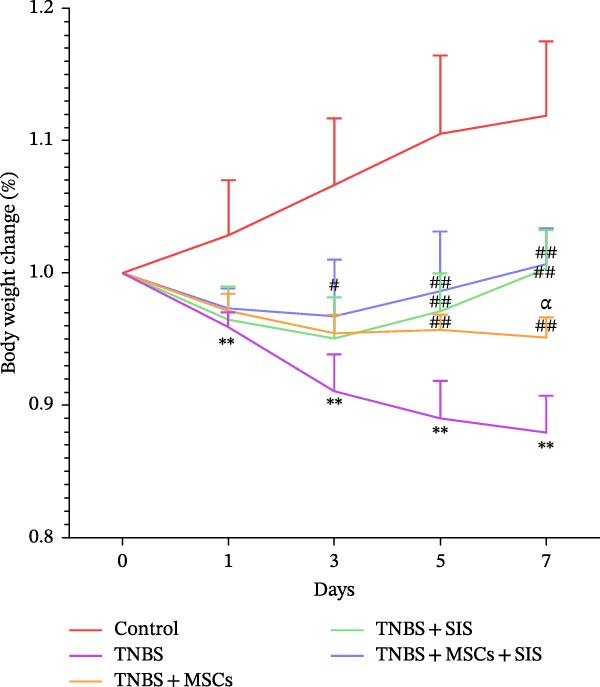
(A)

**Figure:**
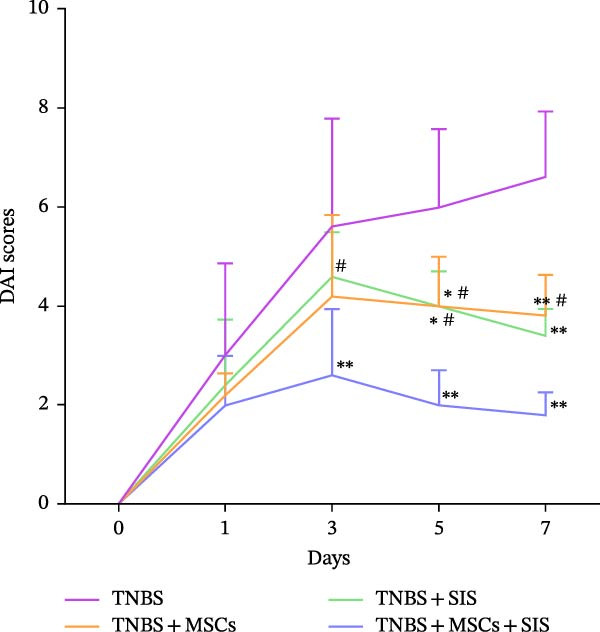
(B)

**Figure:**
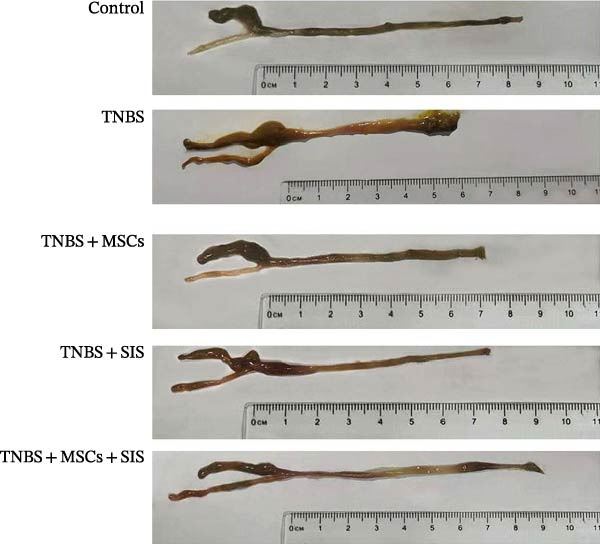
(C)

**Figure:**
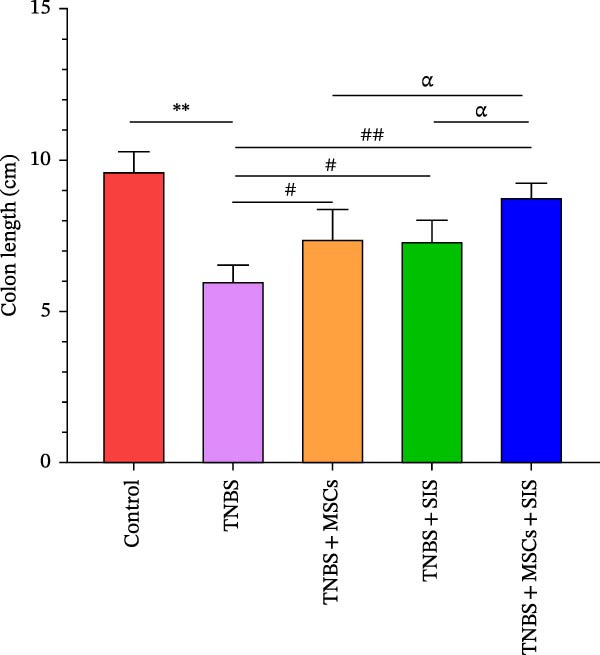
(D)

Differences in weights and DAIs of the mice are shown in Figure 5. Mice in the TNBS group exhibited significant weight loss and higher DAI scores compared to the control group. MSCs and SIS hydrogel had a notable impact on body weight. A great decrease was noticed in the DAI in the colitis group on Day 3, Day 5, and Day 7 of the TNBS + MSCs/SIS group in contrast to the TNBS groups (*p*  < 0.01). The body weight was also evaluated in the TNBS + MSCs/SIS group between Day 5 and Day 7 of colitis induction as compared to the TNBS groups (*p*  < 0.01). What is more, after treatment with BM‐MSCs and SIS hydrogel, significantly elevated body weights and decreased DAI scores were observed compared with the TNBS + MSCs/SIS group. In the mice of the TNBS group, we observed significantly shorter colon lengths compared to the control group. Administration of MSCs/SIS hydrogel notably influenced the colon lengths in the TNBS‐induced colitis mice. The colon length was longer in the MSCs + SIS hydrogel administration group than in the TNBS + MSCs/SIS group.

### 3.6. Microscopic Colitis Scoring

**Figure 7:**
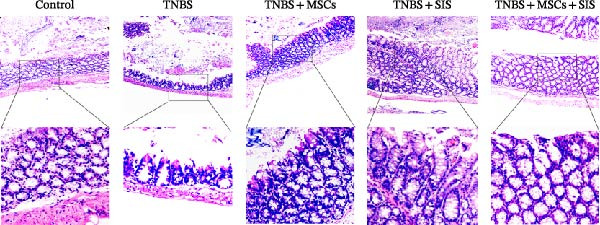
Histological analysis of TNBS‐wounds in the mice (original magnification, 20×).

Histological analysis of colonic tissue revealed severe inflammatory infiltration in the colon of TNBS‐treated mice, as observed through H&E staining. As expected, TNBS mice treated with BM‐MSCs or SIS hydrogel showed decreased infiltration in comparison with TNBS mice. Importantly, those mice treated with BM‐MSCs and SIS hydrogel showed ameliorated inflammation infiltration compared with those treated with BM‐MSCs or SIS hydrogel alone (Figure 7). The experiments confirmed that the combination of BM‐MSCs and SIS hydrogel treatment significantly improved the histology of colitis.

### 3.7. BM‐MSCs and SIS Hydrogel Composite Reduces M1 Macrophage Polarization Markers IL‐1β and TNF‐α Processing in a Serum Biochemical Analysis of the TNBS‐Colitis Mice

To explore the potential medical mechanism of BM‐MSCs and SIS hydrogel in vivo, we detected IL‐1β and TNF‐α, the M1 macrophage polarization markers, in blood serum to examine the inflammatory states in mice. Cytokine levels in serum collected from the hearts of mice in each group were analyzed following anesthesia. As indicated by Figure 8A,B, those mice that have been treated with TNBS in vivo secreted more IL‐1β and TNF‐α in contrast with normal mice ( ^∗∗^ *p* < 0.01).The serum proinflammatory cytokines markedly decreased when they were treated with BM‐MSCs or SIS hydrogel in the TNBS + MSCs group, the TNBS + SIS group, and the TNBS + MSCs + SIS group (^##^ *p* < 0.01). The data indicate that BM‐MSCs and SIS exposure decrease the inflammatory responses in the TNBS‐induced colitis mice and prohibit the polarization of macrophages to M1. What is more, the titers of TNF‐α and IL‐1β levels markedly decreased in the TNBS + MSCs + SIS group in contrast with the TNBS + MSCs group (^ɑɑ^ *p* < 0.01). However, no significant difference was observed between the TNBS + SIS and the TNBS + MSCs + SIS group. The study suggested that SIS hydrogel could play a vital role in assisting BM‐MSCs anti‐inflammatory function. Collectively, these data demonstrated that BM‐MSCs and SIS hydrogel relieve experimental colitis through reducing the secretion levels of IL‐1β, and TNF‐α.

Figure 8Impact of BM‐MSCs and SIS hydrogel composite on cytokine profiles in the TNBS‐treated mice. Blood serum samples was analyzed for IL‐1β (A) and TNF‐α (B) concentrations using one‐way ANOVO. Results are expressed as mean ± SD values.  ^∗∗^ *p* < 0.01 (TNBS vs. control group); ^##^ *p* < 0.01(LPS‐only vs. LPS + MSCs, LPS + SIS or LPS + MSCs + SIS groups) ^ɑɑ^ *p* < 0.01 (LPS + MSCs + SIS group vs. LPS + MSCs group); ns, no statistical significance.

**Figure:**
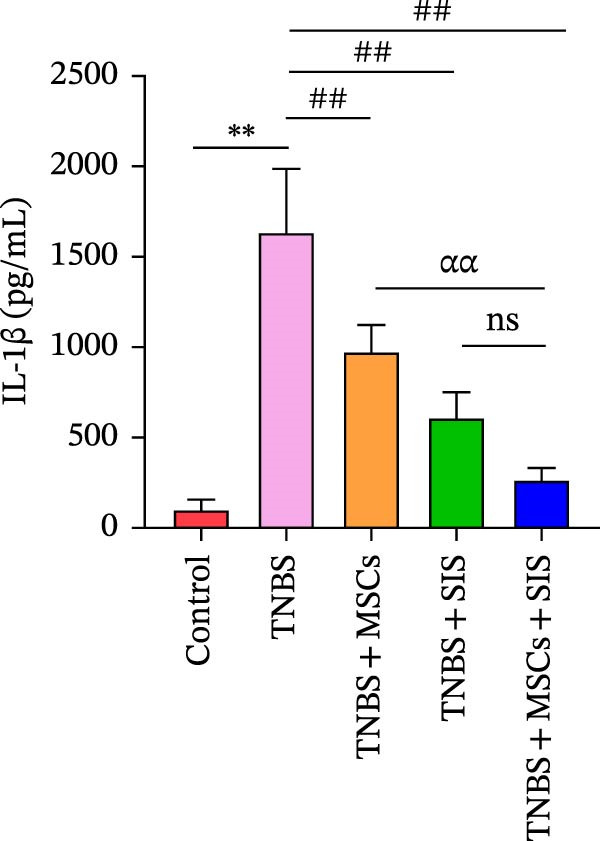
(A)

**Figure:**
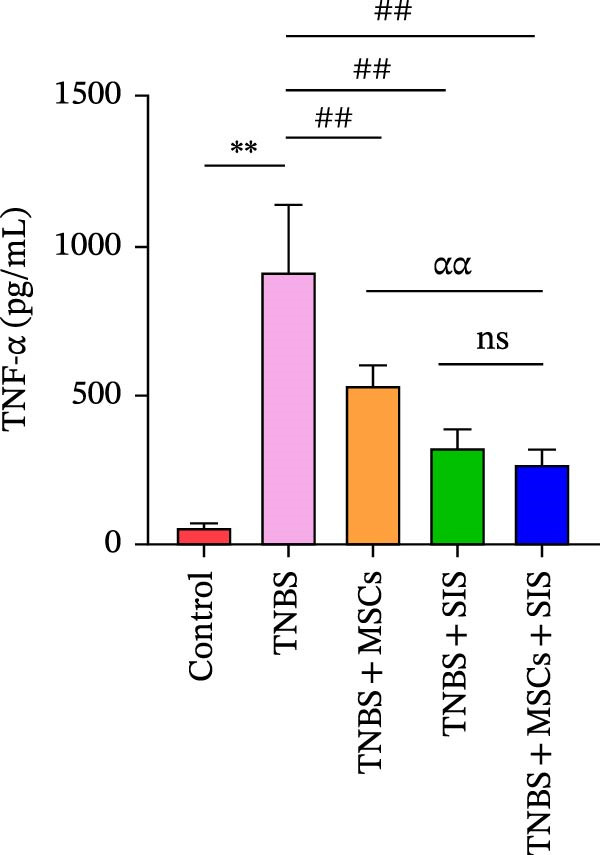
(B)

## 4. Discussion

In the study, the BM‐MSCs and SIS hydrogel composite were used to promote proliferation and investigate their potential use in the management of IBD for better disease control and fewer adverse effects. In my study, we have demonstrated the inhibited M1 macrophage polarization effect of the MSCs and SIS hydrogel composite against inflammation using a mouse model of TNBS‐ and LPS‐stimulated macrophages. We found that TNBS upregulated IL‐1β and TNF‐α in TNBS‐induced mice. Compared with BM‐MSCs, the MSCs and SIS hydrogel composite had a better effect in attenuating the inflammatory reaction induced by TNBS and LPS, in part through the inhibition of M1 macrophage polarization.

As a category of chronic idiopathic disease, IBD features inflammation‐related epithelial barrier damage in the intestinal tract. IBD is a chronic, relapsing condition that severely impacts the gastrointestinal tract, with an unclear etiology and pathology [61]. Current treatments provide only temporary relief, and a definitive cure has yet to be found [62]. Recent studies have indicated that inflammation and infection play key roles in the progression of the disease, frequently leading to damage of the intestinal mucosa and disruption of barrier function [63]. Anti‐inflammatory drugs and biologics are commonly used to manage IBD, but they are associated with lifelong treatment, limited long‐term efficacy, and various side effects [64, 65]. The TNBS‐induced mouse model effectively replicates certain aspects of CD in humans, making it an ideal model for studying intestinal inflammation mechanisms and potential therapies [66–68]. Many studies have reported that high‐level proinflammatory cytokines, macrophage polarization status, and immune‐mediated angiogenesis were fundamental to IBD pathogenesis [69], which is in agreement with our findings. Macrophages are critical in regulating intestinal microenvironment homeostasis and promote inflammation in injured colon tissue through phenotypic polarization. As a result, proinflammatory monocytes are recruited, leading to the release of IL‐1β and TNF‐α, which drive the inflammatory response. This causes disruption of the epithelial barrier, epithelial cell apoptosis, and intestinal inflammation, all of which are closely linked to IBD progression t [20, 70, 71]. Macrophages can be classified into two phenotypes: M1 and M2, with M1 promoting inflammation and M2 preventing it. A balance between them is critical for regulating injury, inflammation, and tissue repair [72, 73]. M1 macrophages can generate high levels of proinflammatory cytokines (IL‐6, IL‐12, and TNF‐α), which in combination with high‐level ROS, can disrupt the intestinal epithelial barrier [74]. CD206 serves as a surface marker for murine and human M2 macrophages induced by IL‐4/IL‐13 or IL‐10 [73]; M2 macrophages can generate high levels of EGF, TGF‐β, IL‐10, and VEGF, while releasing lower levels of IL‐1β, TNF, and IL‐12. These cytokines facilitate angiogenesis and help repair and restore the intestinal epithelial barrier. As inflammation progresses, the regulation mechanism of immunoinflammatory response will turn M1 macrophages to M2 macrophages, which repair the inflammatory damage and inhibit further progression of inflammation [72, 73] As inflammation progresses, the immune response shifts from M1 to M2 macrophages, which aids in tissue repair and limits further inflammation. Furthermore, according to research findings, the inhibition of M1 polarization was able to mitigate the symptoms of colitis [71, 75]. A balance between the functions and phenotypes of these two macrophages is helpful for the determination of the progression of IBD [76, 77].

In this study, colitis was induced in mice by injecting TNBS by enema. Compared with normal mice, TNBS‐treated mice showed significantly decreased body weights, enhanced DAI scores, and notable reduction in colon length. Additionally, TNBS mice showed severe inflammation infiltration in colon tissues from the colon, as evidenced by H&E staining. Meanwhile, cytokine IL‐1β and TNF‐α concentrations in serum also increased. What is more, we observed that the mRNAs expressions of the inflammatory cytokines IL‐6 and TNF‐α (inflammatory cytokines) were significantly higher in the LPS‐induced RAW264.7 in comparison to the control group (*p*  < 0.01).

Recently, MSCs emerged as a potential therapeutic approach for IBD due to their immunoregulatory effects [78]. Previous research has demonstrated that bone marrow‐derived MSCs can help reduce intestinal inflammation and potentially reverse colitis in various experimental models [79]. MSCs were found to demonstrate anti‐inflammatory and immunomodulatory activities by attenuating T‐cell functionality, inhibiting T‐cell proliferation, and reprogramming macrophages to the M2 phenotype [80, 81]. After transplantation, by means of homing to the injured tissues, MSCs were able to repair damaged cells in situ and could also promote immune balance by means of secreting anti‐inflammatory cytokines and chemokines [82]. In a study of Yuan et al. [83], transplantation of ADMSCs could alleviate colon inflammation induced by DSS and inhibit M1 macrophages infiltration. Some other studies have shown that M1 macrophage (induced by GM‐CSF + IFN‐γ or M‐CSF + LPS) could be repolarized by MSCs into IL‐10, which expresses M2 macrophages [84]. In this study, we probed into the effects of MSC therapy using an in vivo animal model and then examined the physiological outcomes of subsequent MSC treatment. We demonstrated that MSCs derived from BALB/c mice promoted the proliferation of Raw264.7 cells in vitro and accelerated the recovery from TNBS‐induced colitis in BALB/c mice in vivo. Following TNBS injection, administration of MSCs by enema was able to effectively accelerate the functional and morphologic recovery and mitigate the severity of TNBS‐induced colitis clinically and pathologically, as shown by certain disease parameters, including acceleration of functional and morphologic recovery, reduced body weight loss, and decreased inflammatory cell infiltration. The clinical DAI score was lower, and colon lengths were longer in the MSCs + LPS‐treated colitis mice compared to the TNBS group. What is more, the titers of the inflammatory mediators, including IL‐1β and TNF‐α, were significantly reduced. In this study, LPS‐induced CD86 and IL4‐induced CD206 were used, and the findings suggested that the CD86 expression could be inhibited and CD206 expression could be enhanced by MSCs. In addition, the mRNA expressions of *IL6* and *TNFα* decreased in the LPS + MSCs group, causing us to hypothesize that MSCs prevented inflammation by inhibiting macrophage polarization towards the M1 phenotype.

The effectiveness of stem cell therapy is determined by the count of surviving cells following the transplantation [38]. Unsatisfactory cell engraftment and retention after transplantation have been considered as the major factors that hinder the promotion of stem cell‐based therapies [37]. Tissue engineering with stem cells as well as biomaterials can be used as a strategy for the preservation and regeneration of tissue in case of an injury [13]. SIS has been explained as a cell‐free, naturally occurring, and collagenous ECM that contains the arrangement of bioactive molecules. SIS is beginning to be used as an acellular and resorbable bioscaffold for tissue repair in different fields [85]. It has been endowed with an appropriate structure and a microenvironment by the special biochemical constituents and brilliant mechanical properties, allowing it to be used as a scaffold. In addition, it also has some physiological functions that are fitting for proliferation, cell adhesion, ultimate tissue regeneration, and differentiation [86–88]. In order to further confirm our findings, SIS hydrogel and MSCs + SIS hydrogel were administered to mice, and the findings implied that the expressions of factors facilitating inflammation, IL‐1β, and TNF‐α, in these mice decreased in contrast with those in the LPS + MSCs group, suggesting that the SIS induction taking place after LPS treatment can be mitigated by the MSCs therapy.

In this study, we showed that the MSCs + SIS hydrogel composite administered via enema for TNBS‐induced colitis repair yielded favorable results, demonstrating greater efficacy than BM‐MSCs or SIS hydrogel alone. The treatment reduced body weight loss and inflammatory cell infiltration. Clinical DAI scores were lower, and colon lengths were longer in the TNBS + MSCs + SIS group compared to the TNBS + MSCs group. Additionally, the levels of the inflammatory mediators IL‐1β and TNFα were significantly reduced.

Moreover, BM‐MSC‐SIS‐CM could stimulate the proliferation of Raw264.7 compared with BM‐MSC‐CM alone (*p*  < 0.01); lower expressions of CD86, *IL6*, and *TNFα* mRNA were found in the LPS + MSCs + SIS group as against the LPS + MSCs group in vitro; however, the CD206 and *IL10* expressions were enhanced in BM‐MSC + SIS treated RAW264.7 cells. These findings suggested that BM‐MSC‐SIS‐CM is more effective at inhibiting M1 macrophage polarization while promoting M2 polarization compared to BM‐MSC‐CM alone.

To sum up, we probed into the mechanism of influence of composite materials on the inflammatory microenvironment regulation in vitro and in vivo. The findings from the experiment of culture medium of the BM‐MSCs‐SIS and BM‐MSCs composite induced by macrophages indicated that the former has a significantly stronger anti‐inflammatory effect compared to the latter.

## 5. Conclusion

According to the findings of this study, we concluded that administration of BM‐MSCs could alleviate colitis by inhibiting M1 macrophage polarization both in vivo and in vitro, which might be facilitated by the SIS hydrogel. Our findings provided a valuable biological hydrogel scaffold to BM‐MSCs for chemical colitis in terms of the regulation of intestinal inflammation and also offered a reference for the formulation of novel therapeutic strategies mediated by macrophages. Nevertheless, it is necessary to carry out studies in patients with IBD in the future so as to thoroughly clarify the mechanism underlying the effects of the SIS hydrogel and BM‐MSC composite on the gut.

## Author Contributions

Jin Huang, Yan Zhou, and Jinjin Fu conceptualized and designed the study and were responsible for drafting the manuscript. Zikai Sun, Xiang Geng, Yu Gong, Fengdong Li, Shaohua Zhuang, and Xiang Cao collected, analyzed, and interpreted the experimental data. Zikai Sun, Shaohua Zhuang, Xiang Cao, and Jinjin Fu made significant revisions to the manuscript to enhance its intellectual content.

## Funding

This study was supported by the Fund of Nanjing Medical University (Grant NMUB2020068), the Basic Research Project of Changzhou Medical Center of Nanjing Medical University (Grant CMCB202332), and the Science and Technology Project of Changzhou Health Commission (Grant QN202313).

## Disclosure

All authors reviewed and approved the final version of the manuscript.

## Ethics Statement

Ethical approval was granted by the Ethics Committee of The Changzhou No. 2 People’s Hospital Affiliated with Nanjing Medical University. Written informed consent was obtained from all participants and/or their guardians.

## Consent

The authors have nothing to report.

## Conflicts of Interest

The authors declare no conflicts of interest.

## Data Availability

The datasets used or analyzed during the current study are available from the corresponding author upon reasonable request.
